# Changes in pre‐haemodialysis serum creatinine levels over 2 years and long‐term survival in maintenance haemodialysis

**DOI:** 10.1002/jcsm.13515

**Published:** 2024-06-18

**Authors:** Seok Hui Kang, Gui Ok Kim, Bo Yeon Kim, Eun Jung Son, Jun Young Do, Jung Eun Lee

**Affiliations:** ^1^ Division of Nephrology, Department of Internal Medicine, College of Medicine Yeungnam University Daegu Republic of Korea; ^2^ Health Insurance Review and Assessment Service Wonju Republic of Korea; ^3^ Department of Medicine, Samsung Medical Center Sungkyunkwan University School of Medicine Seoul Republic of Korea

**Keywords:** haemodialysis, prognosis, protein‐energy wasting, sarcopenia, serum creatinine, survival

## Abstract

**Background:**

Pre‐haemodialysis (HD) serum creatinine levels are reliable and inexpensive markers of muscle mass and important predictors of survival in patients with stable chronic HD. We aimed to assess whether changes in pre‐HD serum creatinine levels during a 2‐year period are linked to long‐term patient survival.

**Methods:**

We retrospectively analysed patients enrolled in a periodic HD quality assessment program. Of the 21 846 participants in the fourth HD quality assessment program, 13 765 were presented in the fifth, of which 10 299 eligible patients were included in this study. We assessed the change in serum creatinine levels over 2 years. The patients were categorized into the following three groups: stable group (patients with change in serum creatinine < 1 mg/dL during 2 years of HD, *n* = 5664), increasing group (patients with increase in serum creatinine ≥ 1 mg/dL, *n* = 2419) and decreasing group (patients with decrease in serum creatinine ≥ 1 mg/dL, *n* = 2216).

**Results:**

The duration of HD at baseline was 62–83 months, with diabetic kidney disease being the most common cause of kidney failure in 36.4% of patients. The 5‐year patient survival rates in the stable, increasing and decreasing groups were 69.1%, 71.3% and 66.8%, respectively. The decreasing group had poorer patient survival than the other two groups (*P* = 0.083 for stable vs. increasing group; *P* = 0.011 for stable vs. decreasing group; *P* < 0.001 for increasing vs. decreasing group). There was no significant difference in the cardiovascular event‐free survival rate among the three groups. Multivariable Cox regression analyses revealed the highest hazard ratio (HR) for mortality in the decreasing group (HR 1.33, 95% confidence interval [CI] 1.21–1.45, *P* < 0.001 vs. stable group; HR 1.50, 95% CI 1.34–1.69, *P* < 0.001 vs. increasing group). The increasing group exhibited a lower risk of mortality than the stable group (HR 0.88, 95% CI 0.81–0.97, *P* = 0.008). Subgroup analyses based on age, HD vintage, sex, Charlson comorbidity index score, presence of diabetes and baseline serum creatinine level tertiles revealed that the decreasing group exhibited the highest mortality among all subgroups.

**Conclusions:**

Our results demonstrate that changes in pre‐HD serum creatinine levels over 2 years of HD were associated with all‐cause mortality in patients undergoing HD. This finding suggests a simple and promising approach for clinicians in the prognosis and management of patients undergoing HD.

## Introduction

End‐stage kidney disease (ESKD) is gaining attention as a chronic condition with its rapidly increasing prevalence, causing a significant burden characterized by high mortality rates and healthcare expenditure.^1^, ^2^ Haemodialysis (HD) is the most common among renal replacement modalities, implemented in over half of the cases worldwide. Although recent technological advancements in HD have contributed to a continuous reduction in short‐term mortality rates among patients undergoing HD, there is growing emphasis on treatment strategies aimed at enhancing long‐term survival.^3^

Sarcopenia and protein‐energy wasting (PEW) are particularly significant conditions because of their widespread occurrence and strong association with mortality.^4^ The assessment of PEW can involve various biomarkers, such as serum albumin or transferrin, anthropometric indicators like body weight, body mass index and waist circumference and radiological/electrical measures, including dual‐energy X‐ray absorptiometry or bioimpedance.^5^ Moreover, a previous study suggested that ultrasound‐based local muscle mass measurement could be a useful option for predicting PEW without exposure to current flow or an X‐ray.^6^ Muscle mass, which is directly measured or indirectly calculated, is often considered the most critical indicator of PEW. However, there is an ongoing debate regarding the currently accepted indicators in clinical practice.

Pre‐HD serum creatinine levels are reliable, inexpensive and easily accessible markers of muscle mass and important predictors of survival in patients with stable chronic HD.^7^ In this study, we aimed to assess whether changes in pre‐HD serum creatinine levels during a 2‐year dialysis period are linked to long‐term patient survival. Our goal was to understand whether alterations in pre‐HD serum creatinine levels, routinely measured in patients undergoing HD, can reflect changes in muscle mass and their implications for the patient's long‐term prognosis. We exclusively included patients who had been on HD for more than 1 year to minimize the influence of residual renal function.

## Methods

### Data source and study population

We conducted a retrospective study of patients enrolled in a periodic HD quality assessment program and examined both program‐related and claims data.^8^, ^9^ South Korea operates under a mandatory single‐payer national healthcare insurance system, covering ~98% of its population. Under this system, patients may be required to pay a portion of the medical expenses for treatments covered by insurance, with the remainder billed by hospitals through the mandatory submission of itemized claims (including fees for procedures, surgeries and prescribed medications) and treatment outcomes (such as continued treatment, transfer, discharge, death or termination of care) to the Health Insurance Review and Assessment Service (HIRA). For uninsured items, patients bear the full cost of treatment without reimbursement from the National Health Insurance Service, although the claims must still be submitted to HIRA. However, patients classified under the medical aid program, due to their low income, submit claims to HIRA without any out‐of‐pocket expenses, with the costs covered by the insurance agency.

Therefore, HIRA possesses comprehensive data on all treatments, procedures, medication prescriptions and outcomes (including mortality) related to the majority of South Korean individuals covered under the National Health Insurance Service. Recently, in response to the rapid increase in the number of patients undergoing HD and treatment costs in Korea, HIRA has been conducting regular evaluations on the quality of care for patients undergoing HD to encourage voluntary quality improvement in hospitals and provide necessary medical information to the public, aiming to promote public health. These evaluations have been conducted every 2 years since their initial introduction in 2009; eight rounds of adequacy assessments have been conducted to date. Each assessment is conducted online, with plans for the adequacy assessment announced in advance to all hospitals performing HD. Patients aged ≥18 years, undergoing HD at least twice a week or at least eight times a month, are targeted for evaluation. During the 6 months of the relative assessment, outpatient cases transferred to other hospitals or admitted cases discharged or transferred to other hospitals are excluded from the evaluation. Hospitals are required to submit lists of included patients and patient data online. The data entry includes indicators of quality management that each HD centre must possess, as selected by HIRA. The data used in this study, from the fourth (July and December 2013) and fifth (July and December 2015) HD quality programs, which included adult patients (aged ≥18 years) undergoing maintenance HD (≥3 months and more than twice weekly), were provided as raw data with anonymized patient and HD centre names. Data on claim records from 1 year before the start of the assessment period to April 2022, along with information on mortality and the date of death, were obtained from HIRA.

**Figure 1:**
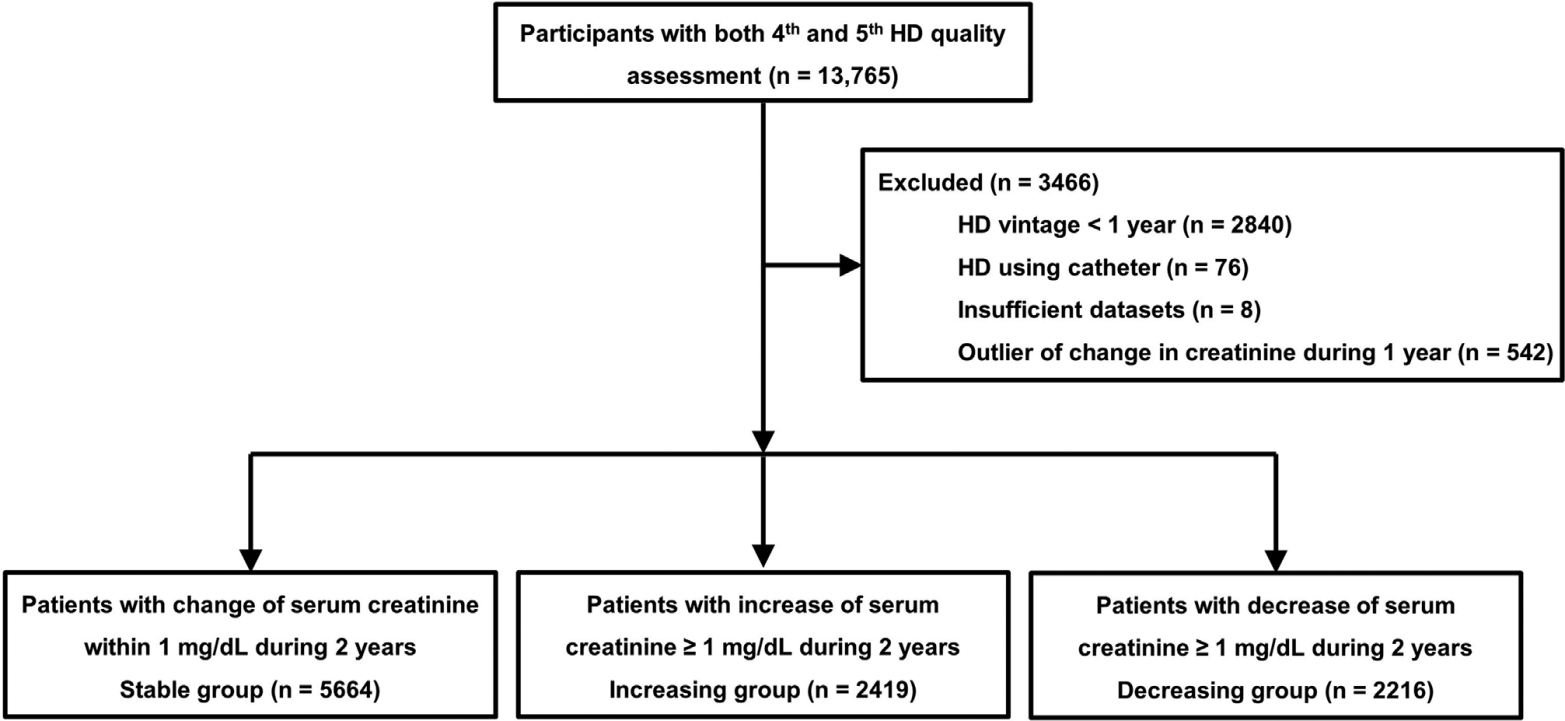
Study flowchart. HD, haemodialysis.

*Figure* *1* presents a flowchart of the study using this dataset. The inclusion criterion was all patients who underwent both fourth and fifth HD quality assessment programs. Of the 21 846 participants in the fourth HD quality assessment program, 13 765 also participated in the fifth program. Among the 13 765 patients, we excluded patients who had HD vintage <1 year in the fourth program, underwent HD via a catheter, had incomplete data or had outliers of change in serum creatinine levels between the fourth and fifth programs (≤2.5 percentile and ≥97.5 percentile). Finally, 10 299 patients were included in this study. This study was approved by the Institutional Review Board of Yeungnam University Medical Center (Approval No. YUMC 2022‐01‐010) and was conducted in accordance with the principles of the Declaration of Helsinki. The requirement for informed consent was waived because patient records and information were anonymized and de‐identified before analysis.

### Study variables

We collected data on age, sex, body mass index, HD vintage (in months), underlying causes of ESKD and the type of vascular access. Haemoglobin levels (g/dL), adequacy of HD treatment (Kt/V_urea_), serum albumin concentration (g/dL), predialysis systolic blood pressure (SBP; mmHg), predialysis diastolic blood pressure (DBP; mmHg), post‐HD body weight, ultrafiltration volume (L per session) and serum calcium, phosphorus and creatinine levels (mg/dL) were recorded as part of the assessment. These data were collected monthly, and all laboratory values were averaged from the monthly collected values. Kt/V_urea_ was calculated using the Daugirdas equation.^10^ The HD quality assessment program recommended monthly initial laboratory findings; however, the program did not specify a particular sample timing, such as short or long inter‐dialytic intervals. Therefore, 8540 (82.9%) patients had samples taken on Mondays or Tuesdays, while the remaining 1759 patients had samples taken on other days.

We calculated the change in serum creatinine levels during the 2 years of the program (serum creatinine value in the fifth program − serum creatinine value in the fourth program). The distribution of changes in serum creatinine levels is shown in *Figure* *S1*. Approximately 50% of patients had a change of <1 mg/dL during those 2 years and were considered to have a stable value. The patients were categorized into the following three groups: stable group (patients with a change in serum creatinine < 1 mg/dL during 2 years of HD), increasing group (patients with an increase in serum creatinine ≥ 1 mg/dL) and decreasing group (patients with a decrease in serum creatinine ≥ 1 mg/dL).

Medications including renin‐angiotensin system blockers (RASBs), aspirin, clopidogrel and statins were evaluated. The medication codes are listed in *Table* *S1*. Medication use was determined based on the identification of one or more prescriptions during the fourth HD quality assessment program. Before assessing HD quality, comorbidities were evaluated for over a year and defined using the Charlson comorbidity index (CCI), which encompasses 17 comorbid conditions.^11^, ^12^, ^13^ The CCI scores were computed for all patients. Additionally, myocardial infarction (MI) or congestive heart failure (CHF) was identified based on the International Classification of Diseases, 10th edition (ICD‐10) codes. We also evaluated the use of diuretics. In South Korea, acetazolamide, torsemide, furosemide, hydrochlorothiazide, chlorthalidone, indapamide, metolazone, amiloride or spironolactone were available. Therefore, we defined the use of diuretics as prescriptions of 30 days or more during 6 months of the fourth HD quality assessment program.

The patients were followed up until April 2022. We evaluated all‐cause mortality as the primary outcome and cardiovascular events (CVEs) as secondary outcomes. HIRA initially confirms the presence or absence of death based on the treatment outcomes reported in the primary claim documents. This information is further validated by verifying whether the patient's insurance has been discontinued within the National Health Insurance Service and the date of the insurance loss. To mitigate errors, HIRA rigorously manages the status of mortality due to the high likelihood of complaints related to overbilling, re‐billing or loss of insurance eligibility. Regarding CVE, it is important to note that certain medications or procedures may not always correlate directly with the occurrence of CVE when ICD‐10 codes are present. In some cases, these may be recorded independent of the actual disease presence or treatment. Considering that our study relies on claim data rather than chart reviews, this results in an inherent limitation. Despite this limitation, research utilizing manipulative definitions based on current claim data is actively pursued. Therefore, we defined CVE as MI, stroke and revascularization regardless of survival or death, as shown in *Table* *S2*.^9^, ^14^, ^15^ In cases where a patient was transferred to peritoneal dialysis or underwent kidney transplantation without experiencing an event, the time of transfer was considered the censoring point.

### Statistical analyses

Data were analysed using SAS Enterprise Guide (Version 7.1; SAS Institute, Cary, NC, USA) and R (Version 3.5.1; R Foundation for Statistical Computing, Vienna, Austria) statistical software packages. Categorical variables are presented as frequencies and percentages, whereas continuous variables are presented as means and standard deviations. We used either Pearson's *χ* ^2^ test or Fisher's exact test to evaluate the statistically significant difference between categorical variables. Differences in continuous variables among the three groups were assessed using one‐way analysis of variance, followed by Tukey's post hoc test. The association between two continuous variables was analysed using Pearson's correlation coefficient. The association between changes in serum creatinine levels and various indicators was evaluated using linear regression analysis.

Survival curves were estimated using the Kaplan–Meier method. *P*‐values for comparison of survival curves were determined using the log‐rank test. Hazard ratios (HRs) and confidence intervals (CIs) were calculated using Cox regression analysis. Multivariable Cox regression analyses were adjusted for age, sex, body mass index, type of vascular access, CCI score, HD vintage, ultrafiltration volume, Kt/V_urea_, haemoglobin, albumin, creatinine, phosphorus and calcium levels, SBP, DBP, use of RASB, aspirin, clopidogrel or statins, presence of MI or CHF, baseline post‐HD body weight and per cent change in body weight over 2 years and were performed using the enter mode. Additionally, subgroup analyses were performed based on age, sex, HD vintage, CCI score, presence of diabetes, tertiles of baseline serum creatinine levels and use of diuretics. Further modification of the Cox regression models was performed using a restricted cubic spline model with 2 df to illustrate the systemic relationship between the change in serum creatinine levels and all‐cause mortality. Statistical significance was set at *P* < 0.05.

For sensitivity analysis, we conducted an additional dataset analysis to validate the relationship between serum creatinine changes and outcomes. Data were collected from patients undergoing HD who had serum creatinine measurements taken at a tertiary hospital (Yeungnam University Medical Center) in January 2017 and January 2019, allowing for the assessment of serum creatinine over a 2‐year period and mortality outcomes. Among the 167 patients with serum creatinine measured in January 2017, we focused on the 99 patients who also had serum creatinine measured in January 2019. Serum creatinine changes were categorized into stable, increasing and decreasing groups based on a threshold of 1 mg/dL, and all‐cause mortality was analysed at the endpoint of follow‐up and determined through chart review.

## Results

### Baseline characteristics

*Table* *1* shows the baseline patient characteristics. In this study, 5664 individuals had serum creatinine levels that remained stable over a 2‐year period (stable group), 2419 individuals experienced an increase of ≥1 (increasing group) and 2216 individuals showed a decrease of ≥1 (decreasing group). The decreasing group had a higher proportion of male patients, longer HD vintages, higher ultrafiltration volumes and higher haemoglobin, phosphorus and creatinine levels than other groups. Additionally, the decreasing group exhibited a lower prevalence of diabetes and lower CCI scores than other groups. The patients in the stable group were older than those in the decreasing group and had a higher Kt/V_urea_ value than the other two groups. The increasing group had lower levels of albumin and calcium than the other two groups. Furthermore, the decreasing group had a lower proportion of statin use and a lower prevalence of MI or CHF than the other two groups. The changes in body mass index in the stable, increasing and decreasing groups during 2 years of HD were −0.03 ± 1.43, 0.15 ± 1.40 and −0.35 ± 1.44 kg/m^2^, respectively (*P* < 0.001).

**Table 1:** Clinical characteristics of patients

	Stable group (*n* = 5664)	Increasing group (*n* = 2419)	Decreasing group (*n* = 2216)	*P*‐value
Age (years)	57.8 ± 12.6	57.7 ± 12.6	57.0 ± 12.6*	0.039
Sex (male, %)	3012 (53.2%)	1405 (58.1%)	1374 (62.0%)	<0.001
Haemodialysis vintage (months)	75.4 ± 60.4	61.6 ± 55.2*	82.5 ± 61.6*, ^+^	<0.001
Body mass index (kg/m^2^)	22.1 ± 3.3	22.4 ± 3.3*	22.3 ± 3.2	<0.001
Haemodialysis sessions per a week				0.026
Two sessions	35 (0.6%)	24 (1.0%)	8 (0.4%)	
Three sessions	5629 (99.4%)	2395 (99.0%)	2208 (99.6%)	
Underlying cause of ESKD				0.034
Diabetes mellitus	2075 (36.6%)	931 (38.5%)	739 (33.3%)	
Hypertension	1589 (28.1%)	670 (27.7%)	674 (30.4%)	
Glomerulonephritis	794 (14.0%)	317 (13.1%)	297 (13.4%)	
Others	494 (8.7%)	197 (8.1%)	204 (9.2%)	
Unknown	712 (12.6%)	302 (12.6%)	302 (13.6%)	
CCI score	6.0 ± 2.6	6.1 ± 2.6	5.8 ± 2.6*, ^+^	<0.001
Vascular access				0.176
Autologous arteriovenous fistula	4994 (88.2%)	2120 (87.6%)	1980 (89.4%)	
Artificial arteriovenous graft	670 (11.8%)	299 (12.4%)	236 (10.6%)	
Kt/V_urea_	1.55 ± 0.27	1.50 ± 0.26*	1.51 ± 0.26*	<0.001
Ultrafiltration volume (L per session)	2.34 ± 1.00	2.24 ± 1.06*	2.53 ± 0.91*, ^+^	<0.001
Haemoglobin (g/dL)	10.7 ± 0.8	10.6 ± 0.8	10.7 ± 0.9*, ^+^	<0.001
Serum albumin (g/dL)	4.03 ± 0.31	4.00 ± 0.33*	4.04 ± 0.31^+^	<0.001
Serum phosphorus (mg/dL)	5.08 ± 1.43	4.96 ± 1.38*	5.48 ± 1.53*, ^+^	<0.001
Serum calcium (mg/dL)	9.02 ± 0.89	8.92 ± 0.90*	9.05 ± 0.99^+^	0.011
Systolic blood pressure (mmHg)	141 ± 16	141 ± 15	141 ± 16	0.521
Diastolic blood pressure (mmHg)	79 ± 9	79 ± 9	79 ± 9	0.032
Serum creatinine (mg/dL)	10.0 ± 2.5	9.1 ± 2.5*	11.5 ± 2.5*, ^+^	<0.001
Use of RASB, *n* (%)	2106 (37.2%)	911 (37.7%)	867 (39.1%)	0.125
Use of aspirin, *n* (%)	2257 (39.8%)	992 (41.0%)	841 (38.0%)	0.100
Use of clopidogrel, *n* (%)	762 (13.5%)	323 (13.4%)	305 (13.8%)	0.911
Use of statins, *n* (%)	1268 (22.4%)	607 (25.1%)	469 (21.2%)	0.004
MI or CHF, *n* (%)	1765 (31.2%)	786 (32.5%)	633 (28.6%)	0.013

*Note*: Data are expressed as mean ± standard deviation for continuous variables and as numbers (percentages) for categorical variables. *P*‐values were tested using one‐way analysis of variance, and Tukey's post hoc test was used for pairwise comparisons. Pearson's *χ* ^2^ test was performed for categorical variables. Abbreviations: CCI, Charlson comorbidity index; CHF, congestive heart failure; ESKD, end‐stage kidney disease; MI, myocardial infarction; RASB, renin‐angiotensin system blocker.

* *P* < 0.05 versus stable group.

^+^ *P* < 0.05 versus increasing group.

### Association between changes in serum creatinine over 2 years of haemodialysis and all‐cause mortality or cardiovascular events

**Figure 2:**
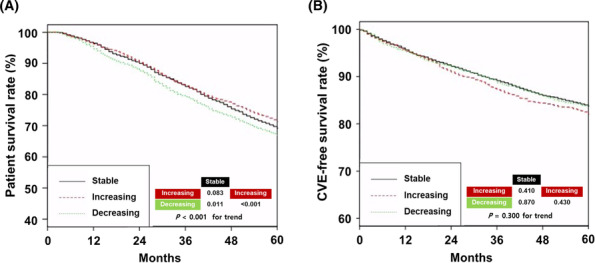
Kaplan–Meier curves based on the three groups. (A) Patient survival rates. (B) Cardiovascular event (CVE)‐free survival rates. *P*‐values for pairwise comparisons with log‐rank tests have been added to the bottom of the graph.

Follow‐up duration in the stable, increasing and decreasing groups was 60 ± 25, 61 ± 25 and 59 ± 26 months, respectively (*P* = 0.004). There were 2122 (37.5%), 852 (35.2%) and 892 (40.3%) deaths in the stable, increasing and decreasing groups, respectively (*P* = 0.002). *Figure* *2* shows the Kaplan–Meier survival curves for the three groups. The 5‐year patient survival rates in the stable, increasing and decreasing groups were 69.1%, 71.3% and 66.8%, respectively (*Figure* *2* *A*; *P* = 0.083 for stable vs. increasing group; *P* = 0.011 for stable vs. decreasing group; *P* < 0.001 for increasing vs. decreasing group). The decreasing group had poorer patient survival than the other two groups. The 5‐year CVE‐free survival rates in the stable, increasing and decreasing groups were 83.7%, 82.1% and 83.7%, respectively (*Figure* *2* *B*). There was no significant difference in the CVE‐free survival rate among the three groups.

Multivariable Cox regression analyses revealed the highest HR for mortality in the decreasing group (*Table* *2*). Additionally, the increasing group exhibited a lower risk of mortality than the stable group (HR 0.88, 95% CI 0.81–0.97, *P* = 0.008). No significant difference was observed in CVE among the three groups in multivariable Cox regression analyses.

**Table 2:** Changes in serum creatinine during 2 years of haemodialysis and the risk of all‐cause mortality or cardiovascular events

	Univariate		Multivariate	
	HR (95% CI)	*P*‐value	HR (95% CI)	*P*‐value
All‐cause mortality				
Ref: Stable group				
Increasing group	0.93 (0.86–1.01)	0.083	0.88 (0.81–0.97)	0.008
Decreasing group	1.11 (1.03–1.20)	0.008	1.33 (1.21–1.45)	<0.001
Ref: Increasing group				
Decreasing group	1.19 (1.09–1.31)	<0.001	1.50 (1.34–1.69)	<0.001
CVE				
Ref: Stable group				
Increasing group	1.10 (0.97–1.24)	0.137	1.09 (0.95–1.25)	0.213
Decreasing group	1.01 (0.89–1.15)	0.864	1.09 (0.94–1.26)	0.236
Ref: Increasing group				
Decreasing group	0.92 (0.80–1.07)	0.287	1.00 (0.84–1.20)	0.981

*Note*: Multivariate analysis was adjusted for age, sex, body mass index, type of vascular access, haemodialysis vintage, Charlson comorbidity index score, ultrafiltration volume, Kt/V_urea_, haemoglobin, serum albumin, serum creatinine, serum phosphorus, serum calcium, systolic blood pressure, diastolic blood pressure, use of renin‐angiotensin system blockers, statin, clopidogrel or aspirin, presence of myocardial infarction or congestive heart failure, post‐haemodialysis body weight and per cent change in body weight and was performed using the enter mode. Abbreviations: CI, confidence interval; CVE, cardiovascular event; HR, hazard ratio.

We performed subgroup analyses based on age (65 years), HD vintage (median value of 51 months), sex, CCI score (median value of 6), presence of diabetes and tertiles of baseline serum creatinine levels (*Table* *S3*). Overall, the decreasing subgroup had the highest mortality among all subgroups. Spline curves also showed that changes in serum creatinine during the 2 years as continuous variables were inversely associated with mortality in both the univariate and multivariable models (*Figure* *S2*). In addition, we performed subgroup analysis based on the use of diuretics. The number of patients without or with diuretics was 4193 (74.0%) and 1471 (26.0%) in the stable group, 1726 (71.4%) and 693 (28.6%) in the increasing group and 1713 (77.3%) and 503 (22.7%) in the decreasing group, respectively. Results using subgroups based on the use of diuretics are shown in *Table* *S4*. Although statistical significance was stronger in patients without diuretics, overall trends were similar to analyses using the total cohort.

### Factors associated with the change in serum creatinine during 2 years of haemodialysis

We analysed the correlation between changes in body weight and serum creatinine levels during 2 years of HD (*Figure* *S3*). Although there was a positive correlation between the two variables, it was notably weak (*r* = 0.162, *P* < 0.001). We further performed linear regression analyses with the changes in serum creatinine levels as the dependent variable (*Table* *3*). An increase in serum creatinine level was positively associated with non‐diabetes mellitus, DBP, RASB use, post‐HD body weight and per cent change in body weight over 2 years. Baseline serum creatinine, female sex, age, HD vintage, CCI score, ultrafiltration volume, Kt/V_urea_ and phosphorus levels were inversely associated with an increase in serum creatinine over 2 years; however, these associations were weak.

**Table 3:** Factors associated with changes in serum creatinine during 2 years of haemodialysis

	Univariate		Multivariate		
	Non‐standardized *β* ± SE	*P*‐value	Non‐standardized *β* ± SE	*P*‐value	VIF	
Baseline serum creatinine	−0.17 ± 0.00	<0.001	−0.24 ± 0.01	<0.001	1.79
Body mass index	−3.80 ± 6.98	0.587	−0.16 ± 6.27	0.980	1.02
Age	0.00 ± 0.00	0.206	−0.02 ± 0.00	<0.001	1.53
Sex (ref: male)	0.06 ± 0.03	0.027	−0.20 ± 0.03	<0.001	1.65
Vascular access type (ref: AVF)	0.05 ± 0.04	0.210	−0.05 ± 0.04	0.218	1.04
Underlying cause of ESKD (ref: DM)	−0.03 ± 0.01	0.006	0.03 ± 0.01	0.001	1.29
Haemodialysis vintage (months)	−0.00 ± 0.00	<0.001	−0.00 ± 0.00	<0.001	1.20
CCI score	0.02 ± 0.01	<0.001	−0.02 ± 0.01	0.001	1.55
Ultrafiltration volume (L per session)	−0.13 ± 0.01	<0.001	−0.08 ± 0.01	<0.001	1.26
Kt/V_urea_	−0.21 ± 0.05	<0.001	−0.25 ± 0.06	<0.001	1.70
Haemoglobin (g/dL)	−0.06 ± 0.02	<0.001	−0.02 ± 0.02	0.165	1.07
Serum albumin (g/dL)	−0.18 ± 0.04	<0.001	−0.01 ± 0.04	0.871	1.15
Serum phosphorus (mg/dL)	−0.12 ± 0.01	<0.001	−0.03 ± 0.01	0.005	1.28
Serum calcium (mg/dL)	−0.07 ± 0.01	<0.001	−0.02 ± 0.01	0.088	1.12
Systolic blood pressure (mmHg)	0.00 ± 0.00	0.486	0.00 ± 0.00	0.588	1.54
Diastolic blood pressure (mmHg)	0.00 ± 0.00	0.359	0.00 ± 0.00	0.008	1.52
Clopidogrel (ref: non‐user)	−0.01 ± 0.04	0.724	−0.06 ± 0.04	0.092	1.10
Aspirin (ref: non‐user)	0.07 ± 0.03	0.014	−0.01 ± 0.03	0.739	1.13
RASB (ref: non‐user)	−0.00 ± 0.01	0.925	0.04 ± 0.02	0.012	1.21
Statins (ref: non‐user)	0.10 ± 0.03	0.002	0.03 ± 0.03	0.408	1.17
MI or CHF	0.06 ± 0.03	0.033	−0.02 ± 0.03	0.498	1.26
Post‐haemodialysis body weight	0.00 ± 0.00	0.260	0.01 ± 0.00	<0.001	1.86
Per cent change of body weight	0.04 ± 0.00	<0.001	0.05 ± 0.00	<0.001	1.05

*Note*: Analyses were performed using linear regression, and all the variables were included as covariates. Abbreviations: AVF, arteriovenous fistula; CCI, Charlson comorbidity index; CHF, congestive heart failure; DM, diabetes mellitus; ESKD, end‐stage kidney disease; MI, myocardial infarction; RASB, renin‐angiotensin system blocker; SE, standard error; VIF, variance inflation factor.

### Sensitivity analysis

In the sensitivity analysis using a single HD centre, the numbers of patients in the stable, increasing and decreasing groups were 57, 30 and 12, respectively. The number of deaths at the endpoint of follow‐up was 16 (28.1%) in the stable group, 9 (30%) in the increasing group and 7 (58.3%) in the decreasing group (*P* = 0.090). The 5‐year survival rates in the stable, increasing and decreasing groups were 69.7%, 66.2% and 38.1%, respectively (*Figure* *S4*) (*P* = 0.205). Due to limited patient numbers, particularly in the decreasing group, statistical significance was not observed. However, a favourable trend in prognosis was observed in the stable or increasing groups, similar to the analysis using the cohort from HD quality assessment programs.

## Discussion

In our study, which examined the long‐term prognosis of patients on maintenance HD based on 2‐year changes in pre‐HD serum creatinine levels, we observed that approximately 25% of patients experienced an increase in serum creatinine levels by ≥1 mg/dL. Further, this group exhibited a lower all‐cause mortality rate than the stable group. In contrast, approximately 25% of patients experienced a decrease in serum creatinine levels by ≥1 mg/dL but had increased mortality rates compared with patients with stable serum creatinine levels. These patterns remained consistent across the various survival and subgroup analyses. Therefore, our findings strongly suggest that changes in pre‐HD creatinine levels can serve as indicators of alterations in muscle mass, providing valuable insights into the presence of PEW. Notably, the relationship between serum creatinine levels and outcomes appeared to be independent of baseline body weight, 2‐year body weight changes and dialysis adequacy. Consequently, serum creatinine levels can prove to be a more reliable and informative marker for assessing PEW, surpassing the utility of body weight alone.

Creatinine level is a surrogate marker of muscle mass.^7^ Guanidoacetate is synthesized from arginine and glycine, and creatine is formed by the transfer of a methyl group from S‐adenosyl‐methionine to guanidoacetate.^16^ The creatine moves into circulation and is actively taken up by muscles. Phosphocreatine or creatine is dehydrated to creatinine, which diffuses into the circulation and is excreted via glomerular filtration or tubular excretion. Therefore, serum creatinine production is strongly associated with muscle mass; however, the creatinine level is strongly influenced by renal clearance and is a well‐known indicator of renal function. Thus, serum creatinine levels may be useful for predicting muscle mass in patients with ESKD who have little residual renal function. Walther et al. evaluated 81 patients on maintenance HD and showed that predialysis creatinine is a useful nutritional indicator, although some creatinine is removed during dialysis.^17^

Our study is novel in its exploration of the changes in serum creatinine levels as a potential indicator of muscle mass variations in patients undergoing HD. While various studies have evaluated serum creatinine levels at a single time point using incident or prevalent HD, evaluating changes over a 2‐point interval is believed to better indicate alterations in muscle mass and nutritional status over a defined period of HD. More importantly, this approach minimizes the impact of age. Grouping based on single‐point serum creatinine levels, regardless of the presence of disease or malnutrition, inevitably introduces age‐related differences in all analyses. However, an approach with a focus on these changes enables a more age‐neutral assessment, providing a variable for observing muscle mass changes over a specific period. In our data, the age difference among the three groups was less than a year, further supporting the benefits of this approach.

Further, our study suggested that changes in serum creatinine levels were not associated with CVE, despite a significant difference in all‐cause mortality. These results may be related to inconsistent findings regarding the association between sarcopenia and mortality reported in previous studies.^4^, ^18^, ^19^, ^20^, ^21^ It is plausible that in patients with sarcopenia, malnutrition may exert a mitigating effect on CVE by reducing fat mass, which potentially decreases the metabolic processes leading to CVE. Our study revealed that patients with a decrease in serum creatinine levels, as opposed to those with stable or increasing levels, exhibited a concurrent decrease in body mass index, which is indicative of a reduction in fat mass. This reduction in fat mass may have contributed to the lower metabolic risk, subsequently reducing the occurrence of CVE. However, despite these findings, when considering the differences in all‐cause mortality, it is likely that non‐CVE‐related factors, such as cachexia and infection, were the primary causes of death. However, the intricate relationship between actual muscle mass reduction and its impact on metabolic‐ and cardiovascular‐specific risks remains unclear, warranting further in‐depth research.

The HD quality assessment program focuses on the overall quality management of HD centres rather than collecting individual patient characteristics. Therefore, it collects the minimal data necessary for quality control. Consequently, data on residual renal function or urine volume were not included in our study, resulting in inherent limitations in completely excluding the influence of residual renal function. Nevertheless, efforts were made to address the impact of this residual renal function exclusion. First, only patients with an HD vintage of >1 year were included in the study, with patients having an average HD vintage of approximately 62–83 months. A previous study using the DOPPS cohort showed that 76.1% of patients undergoing HD for >1 year had a urine volume of <200 mL/day, and this percentage increased to 83.7% for those undergoing HD for over 3 years.^22^ Moreover, Japanese patients undergoing HD showed a residual renal function loss of 86.0% after 1 year of HD and 90.4% after 3 years. Considering these findings, we can assume that significant residual urine volume was unlikely in most patients with an HD vintage of >1 year and an average of 62–83 months. Second, subgroup analysis was performed based on the use of diuretics. Although the use of diuretics cannot be an absolute index of urine volume, it is possible that patients with residual urine or those trying to increase it may be taking diuretics. Conversely, patients who do not take diuretics may either maintain significant urine volume naturally or not have any residual urine volume. However, considering the duration of dialysis, it is expected that a larger proportion of patients without diuretics would be among those without residual renal function. Thus, although statistical significance was stronger in patients without diuretics, overall trends in both subgroups were similar with analyses using the total cohort. Third, the adjustment for ultrafiltration volume may have had some compensatory effects on residual urine volume. Although ultrafiltration volume is influenced by comorbidity and fluid intake, a lower ultrafiltration volume is expected when residual urine is sufficient, while a higher ultrafiltration volume is expected when residual urine is relatively low. In our study, the average ultrafiltration volume per session was approximately 2.2–2.5 L, indicating that a large proportion of patients did not have significant residual renal function. Overall, these considerations may help complement the limitation of not measuring residual urine. However, as the issue of accurate measurement of residual urine has not been completely resolved, further studies targeting only patients without residual urine may lead to more significant results in the future.

In our study, changes in creatinine, independent of body weight, were associated with all‐cause mortality. Recent research has included changes in body weight or body mass index in the diagnostic criteria for PEW. However, serum creatinine levels or their changes are not considered.^23^ Changes in serum creatinine showed only a weak correlation with changes in body weight in our study. Furthermore, when we focused on patients with stable body weight (change <2.5% over 2 years), the association between creatinine changes and all‐cause mortality persisted. These results suggest that changes in creatinine reflect alterations in PEW or muscle mass beyond simple changes in body mass index or body weight and may help predict patient prognosis. Therefore, further studies on the association between changes in creatinine and muscle mass or mortality may provide supporting evidence for including serum creatinine levels or changes as an alternative option related to the diagnosis of PEW in the future.

Nonetheless, our study has some limitations. First, this was a retrospective observational study. The data in our study were originally collected for quality assessment of the HD centre rather than research. However, our dataset may be closer to the real‐world scenario compared with data collected for research from single or multicentre studies. Second, our dataset lacked specific essential data and standardization. In our study, serum creatinine measurement was not specified or standardized. A previous study evaluated 1706 laboratories in South Korea and revealed that more than 88% of centres report serum creatinine using the Jaffe method.^24^ Thus, serum creatinine in most patients would be analysed using the Jaffe method. However, bias based on different or non‐standardized serum creatinine methods may exist as an inherent limitation of our study. Nevertheless, utilizing the changes over two periods may have partially mitigated the errors inherent in the analysis based on a single time point value, assuming that a patient remained on HD at a single centre. In addition, our data did not include residual renal function, although serum creatinine levels in patients undergoing HD are influenced by this factor. As we focused on patients undergoing maintenance HD for over 1 year, we expected the impact of residual renal function to be minimal. Third, we assumed that changes in serum creatinine are an indicator of muscle mass; however, we lack more precise data on changes in body composition measurements or strength, which would provide a more accurate assessment of muscle mass changes. Moreover, such functional evaluations are not commonly included in standard clinical care, which makes their practical application challenging. Fourth, our study did not include data on causes of death. We evaluated CVE using the ICD‐10 code or procedures that assume the occurrence of CVE. However, data on the cause of death may also be useful for establishing a causal relationship. Fifth, the HD quality assessment program recommended monthly initial laboratory findings; however, the program did not specify a particular sample timing, such as short or long inter‐dialytic intervals. Therefore, in our study, the timing of samples taken from each patient did not perfectly align, which could potentially impact serum creatinine levels. Although there were no clear criteria for sample timing and not all cases had consistent sample timing, the fact that samples were taken during the long inter‐dialytic interval in the majority of patients (82.9%) suggests that such discrepancies may not have significantly affected the results. Nevertheless, future studies with consistent sample timing may attenuate the impact of sampling timing and strengthen the clinical impact of the serum creatinine measurements. Sixth, in our study dataset, input regarding dialysis type was not recorded. Additionally, for prescription codes related to HD, the same claim code was used regardless of the type of HD. Therefore, we were unable to obtain information about the type of HD. However, data from the Korean registry, analysed from responses of 65.2% of Korean HD centres in 2013, during which our study was conducted, showed that 86.2% of patients underwent only HD, 3.9% received haemodiafiltration (HDF) once a week, 2.1% received HDF twice a week and 7.9% received HDF three times a week.^25^ Regarding dialysate, 97% used bicarbonate, and over 90% used polysulfone biocompatible dialyzers. In South Korea, due to identical treatment costs between HD and HDF and the lack of data on clear evidence of survival improvement with HDF, most centres primarily perform HD. A similar pattern is observed in the implementation of HD and HDF. Thus, while the type of HD may potentially influence patient survival to some extent, we anticipate that it did not act as a significant confounding factor in our study.

Absolute differences in mortality among the three groups in our study were not large, and statistical significance may have been achieved due to the large sample size alone. In addition, we defined creatinine at each HD quality assessment program as the average creatinine over a period of 6 months and defined patient groups based on the change in mean creatinine over a 2‐year interval. We believe that using such averages and defining patient groups based on changes occurring at predetermined intervals helped overcome variability among individuals. However, our study is fraught with limitations that preclude making definitive conclusions. Therefore, conducting and analysing prospective studies involving standardized sampling and measurement methods for serum creatinine and considering residual renal function while observing creatinine changes would be beneficial. Additionally, collecting data on accurate muscle mass measurements using tools such as bioimpedance, dual‐energy X‐ray absorptiometry and ultrasound in future studies could help demonstrate the impact of creatinine levels or changes on PEW and prognosis in HD patients.

In conclusion, we demonstrated that changes in pre‐HD serum creatinine levels over 2 years of HD were associated with long‐term outcomes in patients undergoing HD. The observed association with mortality rates, independent of baseline body weight and dialysis adequacy, highlights the potential of serum creatinine level as a valuable marker for assessing muscle mass alterations and PEW. This finding suggests a simple and promising approach for clinicians in the prognosis and management of patients undergoing HD.

## Funding

This work was supported by the Medical Research Center Program through the National Research Foundation (NRF) of Korea, funded by the Ministry of Science, ICT, and Future Planning (2022R1A5A2018865), the Basic Science Research Program through the NRF of Korea, funded by the Ministry of Education (2022R1I1A3072966), and the NRF grant funded by the Korea government (MSIT) (2022R1F1A1076151).

## Conflict of interest statement

The authors declare no conflict of interest.

## Supporting information


**Table S1.** Medication types and Health Insurance Review and Assessment Service codes.
**Table S2.** Codes associated with cardiac and cerebrovascular outcomes.
**Table S3.** Subgroup analyses of the association between changes in serum creatinine during 2 years of hemodialysis and all‐cause mortality.
**Table S4.** Cox regression analyses using subgroup analyses based on the use of diuretics.
**Figure S1.** Histogram of changes in serum creatinine during 2 years of hemodialysis.
**Figure S2.** Spline curves for all‐cause mortality according to changes in serum creatinine in patients with < 2.5% change in body weight during 2 years of hemodialysis.
**Figure S3.** The correlation between percent change in body weight and changes in serum creatinine during 2 years of hemodialylsis.
**Figure S4.** Kaplan–Meier curves for patient survival based on the three groups using a cohort of a tertiary medical center.

## Data Availability

The raw data were generated at the Health Insurance Review and Assessment Service. The database can be requested from the Health Insurance Review and Assessment Service by sending a study proposal including the purpose of the study, study design and duration of analysis through the web site (https://www.hira.or.kr). The authors cannot distribute the data without permission.
