# Supplementary material for: Changes in pre‐haemodialysis serum creatinine levels over 2 years and long‐term survival in maintenance haemodialysis
Source: J Cachexia Sarcopenia Muscle. 2024 Jun 18;15(4):1568–77. doi: 10.1002/jcsm.13515 (PMC11294042; doi:10.1002/jcsm.13515)
Supplement: Supplementary file 1 — Table S1. Medication types and Health Insurance Review and Assessment Service codes. Table S2. Codes associated with cardiac and cerebrovascular outcomes. Table S3. Subgroup analyses of the association between changes in serum creatinine during 2 years of hemodialysis and all‐cause mortality. Table S4. Cox regression analyses using subgroup analyses based on the use of diuretics. Figure S1. Histogram of changes in serum creatinine during 2 years of hemodialysis. Figure S2. Spline curves for all‐cause mortality according to changes in serum creatinine in patients with < 2.5% change in body weight during 2 years of hemodialysis. Figure S3. The correlation between percent change in body weight and changes in serum creatinine during 2 years of hemodialylsis. Figure S4. Kaplan–Meier curves for patient survival based on the three groups using a cohort of a tertiary medical center. [file JCSM-15-1568-s001.doc]

**Supplementary materials**

**Table S1.** Medication types and Health Insurance Review and Assessment Service codes

**Table S2.** Codes associated with cardiac and cerebrovascular outcomes

**Table S3.** Subgroup analyses of the association between changes in serum creatinine during 2 years of hemodialysis and all-cause mortality

**Table S4.** Cox regression analyses using subgroup analyses based on the use of diuretics

**Figure S1.** Histogram of changes in serum creatinine during 2 years of hemodialysis

**Figure S2.** Spline curves for all-cause mortality according to changes in serum creatinine in patients with < 2.5% change in body weight during 2 years of hemodialysis

**Figure S3.** The correlation between percent change in body weight and changes in serum creatinine during 2 years of hemodialylsis

**Figure S4.** Kaplan–Meier curves for patient survival based on the three groups using a cohort of a tertiary medical center

**Table S1.** Medication types and Health Insurance Review and Assessment Service codes

| **Medications** | **Codes** |
| --- | --- |
| Alacepril | 104201ATB, 104202ATB |
| Benazepril | 114701ATB |
| Captopril | 122901ATB, 122902ATB, 122903ATB |
| Cilazapril | 133001ATB, 133002ATB, 133003ATB |
| Enalapril | 151601ATB, 151603ATB |
| Fosinopril | 163501ATB, 163502ATB |
| Imidapril | 173401ATB, 173402ATB |
| Moexipril | 196801ATB, 196802ATB |
| Lisinopril | 184501ATB |
| Perindopril | 211301ATB, 211302ATB, 501601ATB, 501602ATB |
| Quinapril | 221901ATB, |
| Ramipril | 222401ATB, 222402ATB, 222404ATB |
| Zofenopril | 510401ATB, 510402ATB, 510403ATB |
| Temocapril | 235002ATB |
| Delapril | 140901ATB, 140902ATB |
| Captopril + Hydrochlorothiazide | 262200ATB, 262300ATB |
| Enalapril + Hydrochlorothiazide | 440300ATB, 453700ATB, 453600ATB |
| Ramipril + Felodipine | 447100ATB, 447200ATB |
| Ramipril + Hydrochlorothiazide | 448600ATB, 448700ATB |
| Perindopril + indapamide | 556200ATB |
| Lisinopril + Hydrochlorothiazide | 499200ATB, 499300ATB |
| Moexipril + Hydrochlorothiazide | 440800ATB, 497900ATB |
| Enalapril + nitrendipine | 466000ATB |
| Candesartan | 122601ATB, 122602ATB, 122603ATB |
| Irbesartan | 177301ATB, 177303ATB |
| Losartan | 185701ATB, 185702ATB |
| Valsartan | 247101ATB, 247102ATB, 247103ATB, 247104ATB |
| Fimasartan | 515201ATB, 515202ATB, 515203ATB |
| Azilsartan | 662401ATB, 662402ATB, 662403ATB |
| Telmisartan | 378801ATB, 378802ATB |
| Eprosartan | 429201ATB |
| Olmesartan | 468501ATB, 468502ATB, 468503ATB, 520901ATB, 520902ATB |
| Valsartan + Amlodipine | 492800ATB, 492900ATB, 495800ATB, 522600ABTB, 522700ABTB, 522800ABTB, 522900ABTB, 523000ATB, 523100ATB, 523200ATB, 523300ATB, 523400ATB |
| Valsartan + Lercanidipne | 522200ATB. 522300ATB. 522400ATB |
| Valsartan + Pitavastatin | 634900ATB, 635000ATB, 635100ATB, 635200ATB |
| Valsartan + Sacubitril | 651401ATB, 651402ATB, 651403ATB |
| Valsartan + Rosuvastatin | 629700ATB, 629800ATB, 525000ATB, 525100ATB, 525200ATB, 525300ATB, |
| Valsartan + Hydrochlorothiazide | 356400ATB, 442600ATB |
| Olmesaetan + Amlodipine | 500500ATB, 500600ATB, 547500ATB. 547600ATB, 547700ATB, 547800ATB, 547900ATB, 548000ATB, 582200ATB, 582400ATB, 629400ATB, 629500ATB, 629600ATB, 631300ATB, 632800ATB, 632900ATB, 633000ATB |
| Olmesartan + Hydrochlorothiazide | 513600ATB |
| Olmesartan + Hydrochlorothiazide + Amlodipine | 519700ATB, 519800ATB, 519900ATB, 520000ATB, 520100ATB |
| Olmesartan + Rosuvastatin | 653200ATB, 644100ATB, 644200ATB, 526300ATB, 526400ATB, 526500ATB, 526900ATB |
| Telmisartan + Hydrochlorothiazide | 502600ATB, 443200ATB, 443300ATB |
| Telmisartan + Rosuvastatin | 629900ATB, 630000ATB, 630100ATB, 630200ATB, 631600ATB, 631700ATB |
| Telmisartan + Amlodipine | 511500ATB, 511600ATB, 511700ATB, 521200ATB, 521300ATB, 521400ATB, 623100ATB, 644800ATB |
| Telmisartan+ Hydrochlorothiazide + Amlodipine | 663500ATB, 663600ATB, 663700ATB, 663800ATB |
| Telmisartan + Rosuvastatin + Amlodipine | 671700ATB, 671600ATB, 671500ATB, 671400ATB, 671300ATB, 671200ATB, |
| Losartan + Hydrochlorothiazide | 262500ATB, 378900ATB, 486900ATB |
| Losartan + Amlodipine | 502700ATB, 503000ATB, 513900ATB, 637400ATB, 637500ATB, 637600ATB |
| Losartan + Rosuvastatin + Amlodipine | 663900ATB, 664000ATB, 664100ATB, 664200ATB, 664300ATB, 664400ATB, |
| Losartan + Chlorthalidone + Amlodipine | 662800ATB, 662900ATB, 663000ATB |
| Fimasartan + Hydrochlorothiazide | 522000ATB, 526800ATB |
| Fimasartan + Amlodipine | 651900ATB, 652000ATB, 652100ATB, 652700ATB, 651900ATB |
| Fimasartan + Rosuvastatin | 654600ATB, 654700ATB, 654800ATB, 654900ATB, 655000ATB |
| Candesartan + Hydrochlorothiazide | 423700ATB |
| Candesartan + Amlodipine | 652900ATB, 653000ATB, 653100ATB, 652900ATB, 652900ATB |
| Candesartan + Rosuvastatin | 673700ATB, 661800ATB, 661900ATB, 662000ATB, 662100ATB |
| Irbesartan + Hydrochlorothiazide | 385700ATB, 385800ATB |
| Irbesartan + Atorvastatin | 527000ATB, 527100ATB, 524000ATB, 524100ATB |
| Azilsartan + Chlorthalidone | 673500ATB, 673600ATB |
| Eprosartan + Hydrochlorothiazide | 460500ATB |
| Atorvastatin + Ezetimibe | 633800ATB, 633900ATB, 634800ATB |
| Pitavastatin + Fenofibrate | 679300ACH |
| Rosuvastatin + Ezetimibe | 640700ATB, 640800ATB, 640900ATB |
| Aspirin | 110701ATB, 110702ATB, 110801ATB, 110802ATB, 111001ACE, 111001ATB, 111001ATE, 111002ATE, 111003ACE, 111003ATE |
| Clopidogrel | 133201ACR, 133201ATB, 133201ATR, 133202ATB, 133203ATR, 506100ATB |
| Aspirin + Bethocarbamol | 256800ATB |
| Aspirin + Clopidogrel | 517900ACH, 517900ACE, 517900ATE, 667500ACE |
| Aspirin + Dipyridamole | 489700ACR |
| Atorvastatin | 111502ATB, 502202ATB, 633900ATB, 472400ATB, 518900ATB, 524100ATB, 527000ATB, 672000ATR, 672100ATR, 111503ATB, 502203ATB, 634800ATB, 472500ATB, 111504ATB, 502204ATB |
| Fluvastatin | 162401ACH, 162402ACH, 162403ATR |
| Lovastatin | 185801ATB |
| Pitavastatin | 470901ATB, 470902ATB, 470903ATB |
| Pravastatin | 216601ATB, 216602ATB, 216603ATB, 216604ATB |
| Rosuvastatin | 454001ATB, 454002ATD, 454002ATB, 454003ATB, 454003ATD, 454005ATB |
| Simvastatin | 227801ATB, 227802ATB, 227803ATB, 227805ATB, 227806ATB |
| Torsemide | 242001ATB, 242002ATB, 242003ATB, 242004ATB |
| Furosemide | 163801ATB, 163802BIJ, 163830BIJ |
| Spironolactone | 231101ATB, 231102ATB |
| Hydrochlorothiazide | 170801ATB |
| Spironolactone + Hydrochlorothiazide | 262700ATB |
| Metoprolol + Hydrochlorothiazide | 262600ATB |
| Eposartan + Hydrochlorothiazide | 460500ATB |
| Bisoprolol + Hydrochlorothiazide | 469800ATB, 469900ATB, 470000ATB |
| Metolazone | 367001ATB, 637002ATB |
| Chlorthalidone | 451301ATB, 451302ATB |
| Indapamide | 174401ATR, 174402ATB, 174403ATB |
| Amiloride | 106901ATB |
| Acetazolamide | 101501ATB, 101502BIJ |

**Table** S2. Codes associated with cardiac and cerebrovascular outcomes

| **ICD-10 codes** |  |
| --- | --- |
| Myocardial infarction | I21–I23 |
| Stroke | I60–I63 |
| **Procedure or operation codes** |  |
| Percutaneous coronary intervention | M6551, M6552, M6561~M6564, M6571, M6572, M6601, M6602 |
| Coronary artery bypass grafting | O1641, O1642, O1647, OA641, OA642, OA647 |
| **Medical treatment codes** |  |
| Protein C | 635801BIJ |
| Tissue type plasminogen activator | 223501BIJ, 223502BIJ |
| Tenecteplase | 450302BIJ, 450301BIJ |
| Tirofiban | 240201BIJ, 240230BIJ |
| Urokinase | 246401BIJ, 246405BIJ, 246407BIJ, 246404BIJ, 246406BIJ |

**Table S3. Subgroup analyses of the association between changes in serum creatinine during 2 years of hemodialysis and all-cause mortality**

|  | **Univariate** | | | **Multivariate** | | | |
| --- | --- | --- | --- | --- | --- | --- | --- |
|  | | **HR (95% CI)** | ***P*-value** | | **HR (95% CI)** | ***P*-value** |  |
| **< 65 years old** | |  |  | |  |  |  |
| Ref: Stable group | |  |  | |  |  |  |
| Increasing group | | 0.91 (0.80–1.03) | 0.126 | | 0.81 (0.70–0.93) | 0.003 |  |
| Decreasing group | | 1.25 (1.12–1.40) | <0.001 | | 1.57 (1.37–1.79) | <0.001 |  |
| Ref: Increasing group | |  |  | |  |  |  |
| Decreasing group | | 1.38 (1.20–1.58) | <0.001 | | 1.94 (1.64–2.30) | <0.001 |  |
| **≥ 65 years old** | |  |  | |  |  |  |
| Ref: Stable group | |  |  | |  |  |  |
| Increasing group | | 0.97 (0.87–1.08) | 0.573 | | 0.97 (0.86–1.10) | 0.625 |  |
| Decreasing group | | 1.11 (0.99–1.23) | 0.064 | | 1.15 (1.01–1.30) | 0.035 |  |
| Ref: Increasing group | |  |  | |  |  |  |
| Decreasing group | | 1.14 (1.00–1.30) | 0.043 | | 1.18 (1.01–1.39) | 0.042 |  |
| **HDV < 51 M** | |  |  | |  |  |  |
| Ref: Stable group | |  |  | |  |  |  |
| Increasing group | | 0.98 (0.88–1.09) | 0.710 | | 0.91 (0.81–1.02) | 0.114 |  |
| Decreasing group | | 1.06 (0.94–1.21) | 0.331 | | 1.24 (1.07–1.43) | 0.004 |  |
| Ref: Increasing group | |  |  | |  |  |  |
| Decreasing group | | 1.09 (0.95–1.25) | 0.242 | | 1.36 (1.15–1.61) | <0.001 |  |
| **HDV ≥ 51 M** | |  |  | |  |  |  |
| Ref: Stable group | |  |  | |  |  |  |
| Increasing group | | 0.82 (0.72–0.93) | 0.002 | | 0.87 (0.75–1.01) | 0.066 |  |
| Decreasing group | | 1.17 (1.05–1.29) | 0.003 | | 1.41 (1.25–1.59) | <0.001 |  |
| Ref: Increasing group | |  |  | |  |  |  |
| Decreasing group | | 1.43 (1.24–1.64) | <0.001 | | 1.62 (1.36–1.92) | <0.001 |  |
| **Male sex** | |  |  | |  |  |  |
| Ref: Stable group | |  |  | |  |  |  |
| Increasing group | | 0.89 (0.80–0.99) | 0.030 | | 0.87 (0.77–0.97) | 0.017 |  |
| Decreasing group | | 1.06 (0.96–1.17) | 0.248 | | 1.38 (1.22–1.55) | <0.001 |  |
| Ref: Increasing group | |  |  | |  |  |  |
| Decreasing group | | 1.19 (1.06–1.24) | 0.004 | | 1.59 (1.37–1.84) | <0.001 |  |
| **Female sex** | |  |  | |  |  |  |
| Ref: Stable group | |  |  | |  |  |  |
| Increasing group | | 0.97 (0.86–1.10) | 0.673 | | 0.90 (0.77–1.04) | 0.135 |  |
| Decreasing group | | 1.15 (1.02–1.31) | 0.027 | | 1.27 (1.10–1.48) | 0.001 |  |
| Ref: Increasing group | |  |  | |  |  |  |
| Decreasing group | | 1.19 (1.02–1.38) | 0.028 | | 1.42 (1.18–1.72) | <0.001 |  |
| **CCI score < 6** | |  |  | |  |  |  |
| Ref: Stable group | |  |  | |  |  |  |
| Increasing group | | 0.90 (0.80–1.02) | 0.092 | | 0.83 (0.73–0.96) | 0.011 |  |
| Decreasing group | | 1.20 (1.08–1.34) | 0.001 | | 1.38 (1.21–1.57) | <0.001 |  |
| Ref: Increasing group | |  |  | |  |  |  |
| Decreasing group | | 1.33 (1.16–1.52) | <0.001 | | 1.66 (1.40–1.96) | <0.001 |  |
| **CCI score ≥ 6** | |  |  | |  |  |  |
| Ref: Stable group | |  |  | |  |  |  |
| Increasing group | | 0.94 (0.84–1.04) | 0.217 | | 0.93 (0.82–1.05) | 0.221 |  |
| Decreasing group | | 1.09 (0.97–1.22) | 0.141 | | 1.27 (1.11–1.45) | <0.001 |  |
| Ref: Increasing group | |  |  | |  |  |  |
| Decreasing group | | 1.16 (1.02–1.33) | 0.024 | | 1.37 (1.17–1.61) | <0.001 |  |
| **DM** | |  |  | |  |  |  |
| Ref: Stable group | |  |  | |  |  |  |
| Increasing group | | 0.94 (0.85–1.05) | 0.305 | | 0.95 (0.84–1.07) | 0.391 |  |
| Decreasing group | | 1.19 (1.06–1.33) | 0.002 | | 1.31 (1.15–1.49) | <0.001 |  |
| Ref: Increasing group | |  |  | |  |  |  |
| Decreasing group | | 1.26 (1.11–1.44) | <0.001 | | 1.39 (1.18–1.63) | <0.001 |  |
| **Non-DM** | |  |  | |  |  |  |
| Ref: Stable group | |  |  | |  |  |  |
| Increasing group | | 0.89 (0.79–0.99) | 0.041 | | 0.85 (0.74–0.98) | 0.020 |  |
| Decreasing group | | 1.11 (0.99–1.24) | 0.052 | | 1.35 (1.19–1.54) | <0.001 |  |
| Ref: Increasing group | |  |  | |  |  |  |
| Decreasing group | | 1.26 (1.10–1.44) | <0.001 | | 1.59 (1.34–1.88) | <0.001 |  |
| **Low-tertile of BCr** | |  |  | |  |  |  |
| Ref: Stable group | |  |  | |  |  |  |
| Increasing group | | 0.82 (0.74–0.91) | <0.001 | | 0.92 (0.82–1.03) | 0.144 |  |
| Decreasing group | | 1.51 (1.30–1.75) | <0.001 | | 1.43 (1.22–1.69) | <0.001 |  |
| Ref: Increasing group | |  |  | |  |  |  |
| Decreasing group | | 1.84 (1.57–2.15) | <0.001 | | 1.57 (1.31–1.88) | <0.001 |  |
| **Mid-tertile of BCr** | |  |  | |  |  |  |
| Ref: Stable group | |  |  | |  |  |  |
| Increasing group | | 0.83 (0.71–0.97) | 0.019 | | 0.86 (0.72–1.03) | 0.094 |  |
| Decreasing group | | 1.62 (1.43–1.85) | <0.001 | | 1.46 (1.26–1.70) | <0.001 |  |
| Ref: Increasing group | |  |  | |  |  |  |
| Decreasing group | | 1.96 (1.65–2.32) | <0.001 | | 1.70 (1.39–2.10) | <0.001 |  |
| **High-tertile of BCr** | |  |  | |  |  |  |
| Ref: Stable group | |  |  | |  |  |  |
| Increasing group | | 0.73 (0.58–0.92) | 0.008 | | 0.77 (0.59–0.99) | 0.045 |  |
| Decreasing group | | 1.25 (1.08–1.45) | 0.003 | | 1.20 (1.02–1.43) | 0.032 |  |
| Ref: Increasing group | |  |  | |  |  |  |
| Decreasing group | | 1.71 (1.35–2.16) | <0.001 | | 1.57 (1.19–2.08) | 0.001 |  |

Multivariate analysis was adjusted for age, sex, body mass index, type of vascular access, HDV, CCI, ultrafiltration volume, Kt/V_urea_, hemoglobin, serum albumin, BCr, serum phosphorus, serum calcium, systolic blood pressure, diastolic blood pressure, use of renin-angiotensin system blockers, statin, clopidogrel, or aspirin, presence of myocardial infarction or congestive heart failure, post-dialysis body weight, and percent change of body weight, and was performed using the enter mode.

**Abbreviations**: BCr, baseline creatinine; CCI, Charlson Comorbidity Index; CI, confidence interval; DM, diabetes mellitus; HDV, hemodialysis vintage; HR, hazard ratio; M, months.

**Table S4. Cox regression analyses using subgroup analyses based on the use of diuretics**

|  | **Univariate** | | | **Multivariate** | | | |
| --- | --- | --- | --- | --- | --- | --- | --- |
|  | | **HR (95% CI)** | ***P*-value** | | **HR (95% CI)** | ***P*-value** |  |
| **Diuretics (-)** | |  |  | |  |  |  |
| Ref: Stable group | |  |  | |  |  |  |
| Increasing group | | 0.92 (0.84–1.01) | 0.091 | | 0.85 (0.76–0.95) | 0.005 |  |
| Decreasing group | | 1.12 (1.03–1.23) | 0.012 | | 1.37 (1.23–1.52) | <0.001 |  |
| Ref: Increasing group | |  |  | |  |  |  |
| Decreasing group | | 1.22 (1.09–1.37) | <0.001 | | 1.61 (1.40–1.85) | <0.001 |  |
| **Diuretics (+)** | |  |  | |  |  |  |
| Ref: Stable group | |  |  | |  |  |  |
| Increasing group | | 0.94 (0.82–1.08) | 0.400 | | 0.93 (0.80–1.10) | 0.408 |  |
| Decreasing group | | 1.11 (0.95–1.29) | 0.200 | | 1.23 (1.03–1.48) | 0.025 |  |
| Ref: Increasing group | |  |  | |  |  |  |
| Decreasing group | | 1.17 (0.98–1.40) | 0.073 | | 1.32 (1.06–1.64) | 0.013 |  |

Multivariate analysis was adjusted for age, sex, body mass index, type of vascular access, hemodialysis vintage, Charlson Comorbidity Index, ultrafiltration volume, Kt/V_urea_, hemoglobin, serum albumin, baseline creatinine, serum phosphorus, serum calcium, systolic blood pressure, diastolic blood pressure, use of renin-angiotensin system blockers, statin, clopidogrel, or aspirin, presence of myocardial infarction or congestive heart failure, post-dialysis body weight, and percent change of body weight, and was performed using the enter mode.

**Abbreviations**: CI, confidence interval; HR, hazard ratio.

**
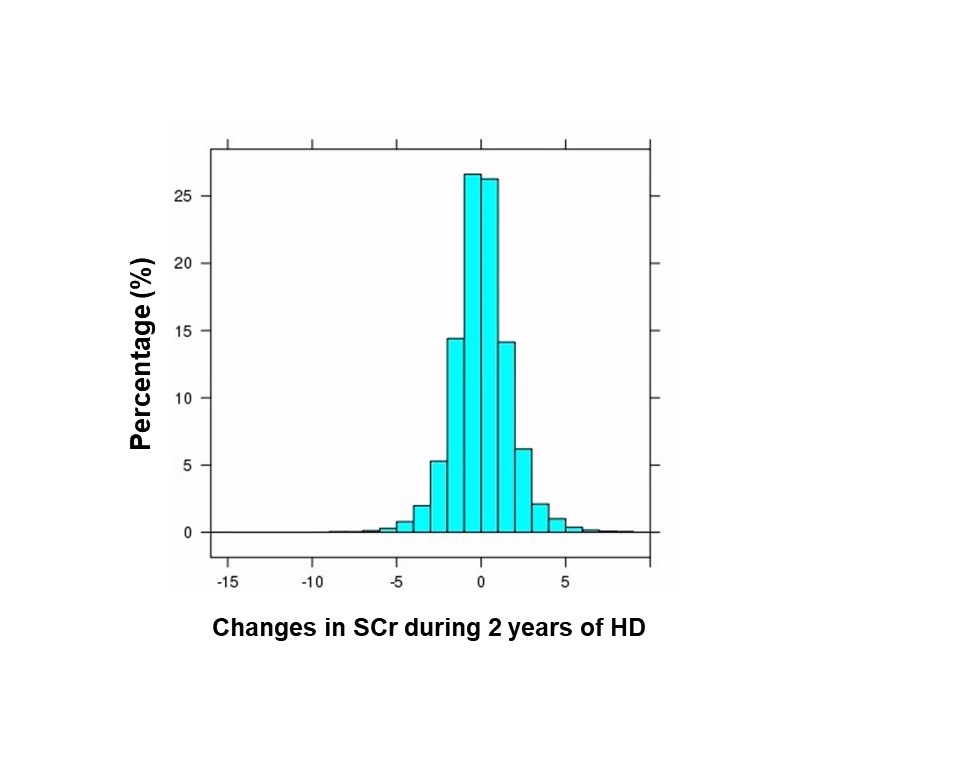
**

**Figure S1. Histogram of changes in serum creatinine during 2 years of hemodialysis.** Abbreviations: SCr, serum creatinine; HD, hemodialysis

**
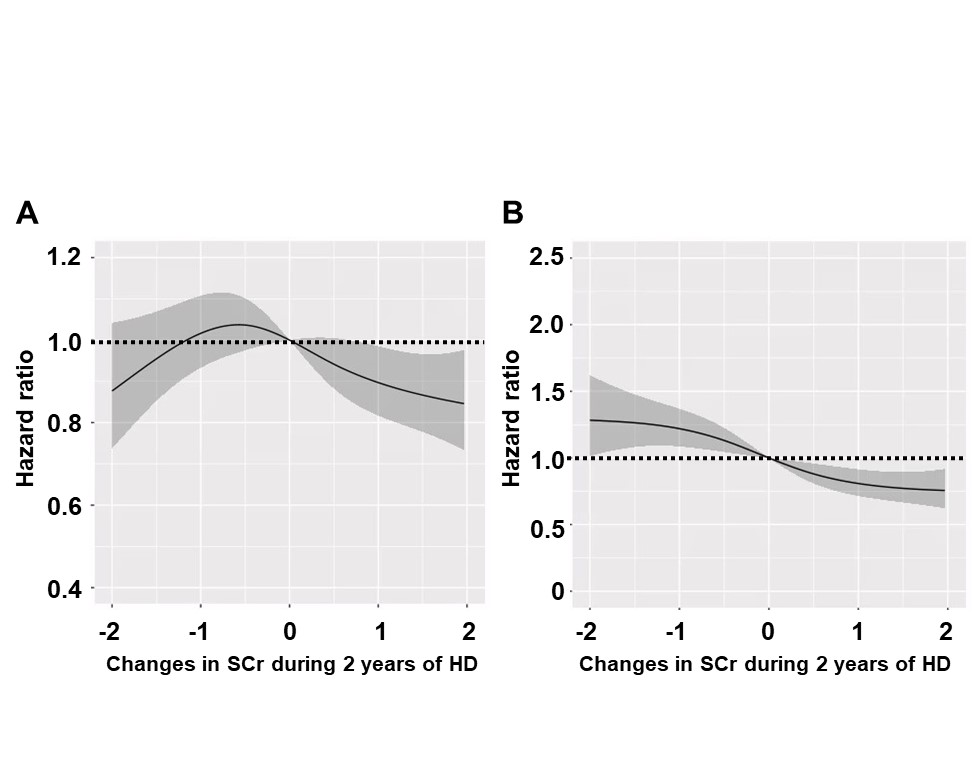
**

**Figure S2. Spline curves for all-cause mortality according to changes in serum creatinine in patients with < 2.5% change in body weight during 2 years of hemodialysis** (A) Univariate model. (B) Multivariate model adjusted for age, sex, body mass index, type of vascular access, hemodialysis vintage, Charlson Comorbidity Index score, ultrafiltration volume, Kt/V_urea_, hemoglobin, serum albumin, serum creatinine, serum phosphorus, serum calcium, systolic blood pressure, diastolic blood pressure, use of renin-angiotensin system blockers, statins, clopidogrel or aspirin, presence of myocardial infarction or congestive heart failure, post-dialysis body weight, and percent change in body weight. Abbreviations: HD, hemodialysis; SCr, serum creatinine

**
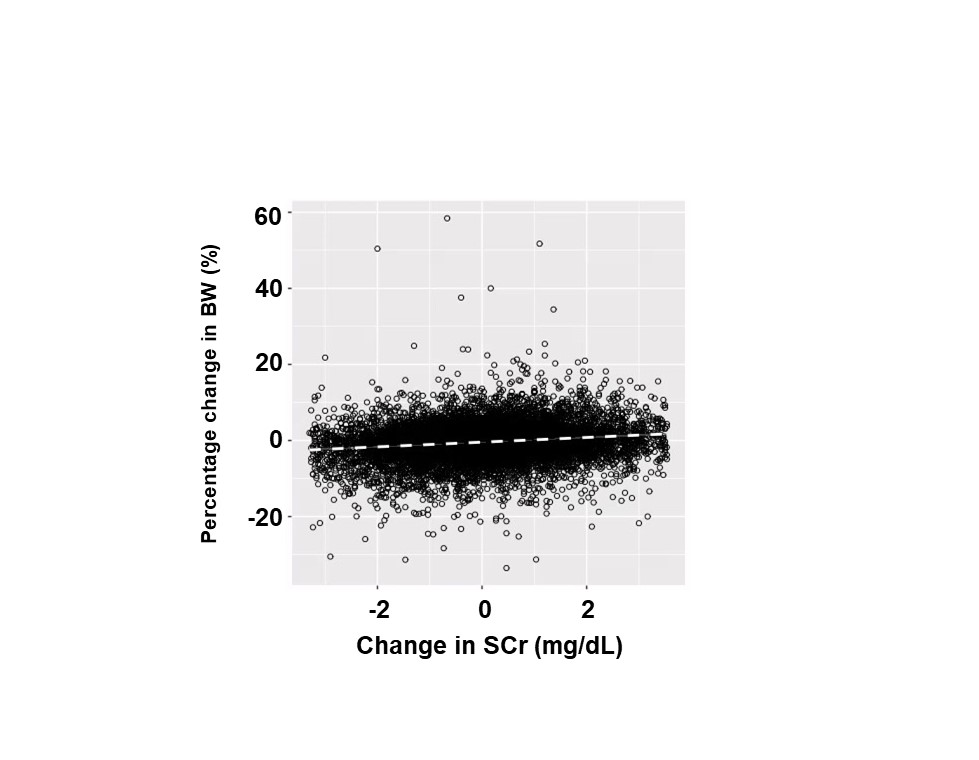
**

**Figure S3. The correlation between percent change in body weight and changes in serum creatinine during 2 years of hemodialysis** (correlation coefficient, 0.162; *P* < 0.001). Abbreviations: BW, body weight; SCr, serum creatinine.

**
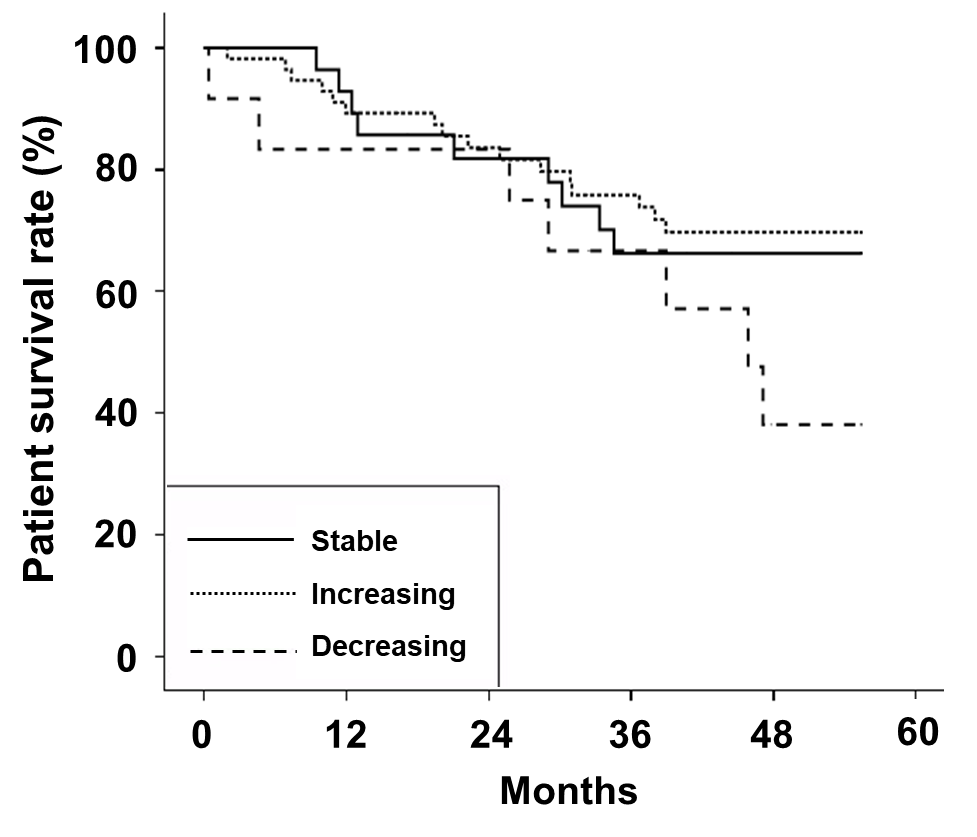
**

**Figure S4. Kaplan**–**Meier curves for patient survival based on the three groups using a cohort of a tertiary medical center.**
